# Clinical status, biochemical profile and management of a single cohort of patients with arginase deficiency

**DOI:** 10.1002/jmd2.12266

**Published:** 2021-12-30

**Authors:** Nandaki Keshavan, Michelle Wood, Lucy M. Alderson, Mario Cortina‐Borja, Rachel Skeath, Mel McSweeney, Marjorie Dixon, Maureen A. Cleary, Emma Footitt, Spyros Batzios

**Affiliations:** ^1^ Department of Paediatric Metabolic Medicine Great Ormond Street Hospital NHS Trust London UK; ^2^ UCL Great Ormond Street Hospital Institute of Child Health London UK; ^3^ Department of Physiotherapy Great Ormond Street Hospital NHS Trust London UK; ^4^ Population, Policy and Practice Research and Teaching Department UCL Great Ormond Street Hospital Institute of Child Health London UK; ^5^ Department of Dietetics Great Ormond Street Hospital NHS Trust London UK

**Keywords:** arginase deficiency, hyperammonaemia, metabolic decompensation, trial end points, urea cycle disorder

## Abstract

Arginase deficiency is a rare autosomal recessive urea cycle disorder (UCD) caused by mutations in the *ARG1* gene encoding arginase that catalyses the hydrolysis of arginine to ornithine and urea. Patients have hyperargininaemia and progressive neurological impairment but generally suffer fewer metabolic decompensations compared to other UCDs. The objective is to describe the clinical features, biochemical profile, neuroradiological findings and experience of managing children with arginase deficiency. Twenty‐year retrospective review of patient medical records at a single metabolic centre was performed. Six patients from three unrelated families were identified. Mean age at first symptom was 3.3 (1.5–9.0) years, while mean age at diagnosis was 8.8 (0.16–15.92) years. Four patients developed spastic diplegia and two of six with spastic quadriplegia with classical features including hyperreflexia, clonus and toe walking. This resulted in gait abnormalities that have been monitored using the GAITRite system and required Achilles tendon release in five children. Generalised tonic‐clonic seizures and/or absences were present in three of six children and were controlled with anticonvulsants. All patients had moderate learning difficulties. Neuroimaging showed cerebral/cerebellar atrophy in four patients and basal ganglia abnormalities in two. Arginine levels were universally elevated throughout follow‐up despite protein restriction, essential amino acid supplementation and ammonia scavengers, and neurological outcome was generally poor. Two patients died following severe metabolic decompensation in adolescence. Children with arginase deficiency continue to present a management challenge of what appears to be an inexorable course of neurocognitive impairment. Further insight into disease mechanisms may provide insight into novel treatment strategies.

**Synopsis**

Paediatric arginase deficiency continues to present a considerable challenge to management. Despite drug therapy and dietary intervention, arginine levels remain persistently elevated and patients suffer recurrent metabolic decompensations and insidiously progressive neurodisability.

## 1 INTRODUCTION

Arginase deficiency (OMIM 207800) is a rare autosomal recessive disorder of protein catabolism. Mutations in the *ARG1* gene result in loss of function of the enzyme arginase that catalyses the conversion of arginine to ornithine and urea. Arginase deficiency causes hyperammonaemic decompensations but may be differentiated from other urea cycle defects by the presence of hyperargininaemia and slowly evolving neurodisability characterised by a combination of limb spasticity, cognitive impairment, learning difficulty and generalised/absence seizures.^1^ The pathophysiological mechanisms underlying the disease are unclear, although neurological impairment is hypothesised to result from shunting of excess arginine to neurotoxic/epileptogenic guanidino compounds via arginine‐glycine amidinotransferase,^2^, ^3^ demyelination of the corticospinal tracts via inhibition of transketolase^4^ and disturbed nitric oxide homeostasis by induction of inducible nitric oxide synthase and generation of superoxide/peroxynitrite free radicals.^2^ In the long term, patients may develop liver fibrosis, cirrhosis and hepatocellular carcinoma.^5^, ^6^

Presently, there are no licenced disease‐modifying treatments for arginase deficiency. Liver transplant reverses biochemical abnormalities^7^ but does not reverse neurological impairment while red cell transfusion‐based enzyme replacement has not demonstrated sustained clinical benefit.^8^ While clinical trials of enzyme replacement therapy (AEB1102, pegzilarginase) are being undertaken currently,^9^ the mainstay of management includes protein restriction and ammonia scavengers. Regular physiotherapy, tendon/contracture release surgery and antispasmodics are useful in maintaining good functional ability.

As the predominant neurological feature is lower limb spasticity, regular assessment of gait is needed. Subjective analysis of gait by observation alone is non‐quantitative and insufficient to monitor patient progress. A more objective means of assessing gait is needed particularly since neurological impairment may evolve insidiously. The GAITRite provides a more reliable, reproducible and quantitative assessment of gait.^10^, ^11^

We describe our experience managing six patients with arginase deficiency and discuss cases where management has been complicated by the presence of a second, otherwise unrelated diagnosis. We also discuss the application of the GAITRite to detect neurological impairment and monitor progress, making it a possible candidate outcome measure in clinical trials of novel therapies.

## 2 METHODS

We retrospectively reviewed the medical notes of all patients with a diagnosis of arginase deficiency managed at our tertiary metabolic centre from 1998 to 2018. Diagnosis of arginase deficiency was based on the presence of qualifying clinical features accompanied by laboratory features including at least one of (1) elevated arginine levels, (2) reduced red cell arginase activity and (3) genetic confirmation of biallelic pathogenic variants in the *ARG1* gene. Data regarding clinical features, biochemistry, neuroradiological findings, GAITRite assessment and patient management were recorded.

Levels of ammonia, arginine, essential amino acids (EAAs) and ornithine were followed up over time, and mean levels deduced from each using area under curve calculations were used. A metabolic decompensation was defined as an episode of presenting unwell with evidence of hyperammonaemia with or without encephalopathy.

The GAITRite system is an electronic carpeted walkway/mat embedded with pressure‐activated mechanical sensors. Patients were asked to walk barefoot at their preferred speed in four consecutive passes. The assessment is performed with the child walking independently; however, two patients were unable to walk on their own and required assistance. During walking, sensors register the position and pressure of each footfall. Velocity, cadence, step length and base of support data from patients were compared with normative data standardised for sex and age.^12^

## 3 RESULTS

### 3.1 Patient cohort

Table 1 summarises the clinical characteristics of the patients. Case histories are detailed in the supplementary file. Mean age at first symptom was 3.3 (range: 1.5–9.0) years, while mean age at diagnosis was 8.8 (0.16–15.92), implying a mean delay to diagnosis of 6.06 (0–11.5) years. All patients met the aforementioned diagnostic criteria. Additionally, Patient 2.1 had reduced arginase activity on red cells. Confirmatory Sanger sequencing was carried out in three cases. Patients 2.1/2.2 were homozygous for c.646_649delCTCA; p.Leu216Alafs*4 and Patient 3 was homozygous for c.93_93delG, p.Arg32Glufs*16. Both mutations have been reported before.^13^, ^14^ Genetic testing was not carried out in siblings of Family 1. Consanguinity was seen in Families 1 and 3. The parents of Family 2 were not known to be consanguineous.

**TABLE 1:** Summary of key clinical features found in the cohort of our patients

ID	Sex	Ethnicity	Age first symptoms/years	Age diagnosis/years	Latency to diagnosis/ years	Age last follow‐up	Basis of diagnosis	Genetics	Neurological features	Cognitive	Seizures	Decompensations over period of follow‐up	Neuroimaging	Treatment
1.1	F	Somali	5	15.92	10.92	28	Clinical features, elevated Arginine	ND	Spastic diplegia	Moderate learning difficulty, IQ < 1st centile	GTC, myoclonic jerks	0	Normal	PR, EAA, Na B, LV
1.2	F	Somali	2.5	11.83	9.33	15.4 (D)	Clinical features, elevated Arginine	ND	Spastic quadriplegia	Moderate learning difficulty and speech delay requiring special school	GTC, absences	2	Cerebral, cerebellar atrophy. MRS: raised lactate peak and low creatine and NAA peaks which are features of infarction.	PR, EAA, Na B, V, C
1.3	M	Somali	1.5	13	11.5	19 (D)	Clinical features, elevated Arginine	ND	Spastic quadriplegia	Cognitively impaired	GTC	3	Pontocerebellar atrophy, bilateral asymmetric regions of cortical atrophy.	PR, EAA, Na B, V, C
2.1	F	Somali	1.5	2	0.5	16.6	Clinical features, enzymology, genetics	c.646_649delCTCA; p.Leu216Argfs*4homozygous	Spastic diplegia	Moderate learning difficulty requiring special school	None	6	Cerebral, cerebellar atrophy	PR, EAA, Na B
2.2	F	Somali	0.16	0.16	0	12	Clinical features, genetics	c.646_649delCTCA; p.Leu216Argfs*4 homozygous	Spastic diplegia	Mild learning difficulty, needing special 1:1 help at mainstream school	None	4	Cerebral, cerebellar atrophy	PR, EAA, Na B
3	M	Pakistani	9	10.16	4.16	14	Clinical features, genetics	c.93_93delG; p.Arg32Glufs*16 homozygous	Spastic diplegia	Moderate learning difficulty, IQ < 0.1 centile, special school	GTC, absences	3	ND	PR, EAA, Na B, V

Abbreviations: C, Carbamazepine; D, deceased; EAA, essential amino acids; GTC, generalised tonic‐clonic; LV, Levetiracetam; NAA, N‐acetyl aspartate; Na B, sodium benzoate; ND, not done; PR, protein restriction; V, sodium valproate.

### 3.2 Neurological features

All patients had significant neurodisability. Spasticity was the most prominent finding on clinical examination including spastic diplegia in four cases and spastic quadriplegia in two. Four patients developed seizures of varying subtype including generalised tonic‐clonic, absences and myoclonic jerks. All patients had some degree of learning difficulty and required educational input, which are detailed in Table 1.

MRI brain findings are summarised in Supplementary Figure 1. Generalised cerebral and cerebellar atrophy were seen. Patient 1.3 developed pontocerebellar atrophy and extensive cortical grey‐matter injury bilaterally leaving asymmetric regions of cortical atrophy.

### 3.3 Growth

Longitudinal growth data were available for Patients 2.1, 2.2 and 3. Supplementary Figure 2 illustrates growth trajectories expressed as height and weight standard deviation score (SDS) against age. Whereas height and weight were normal in early childhood, in all cases, there was a clear decline in growth to below −2 SDS in adolescence.

### 3.4 Biochemical data

**FIGURE 1:**
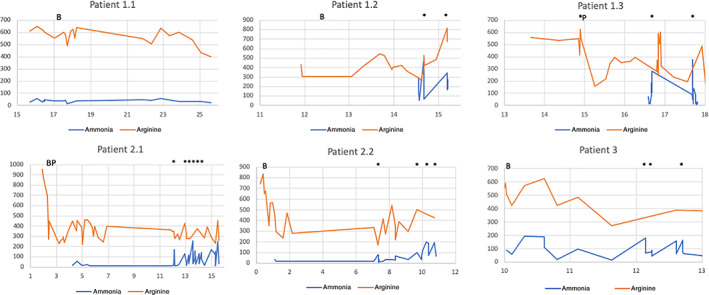
Biochemical data for the six patients. Arginine and ammonia levels (umol/L) followed up over time (age/years). For Patients 2.1 and 2.2, there are no data points between the ages of 7–12 years and 2–7 years, respectively, as they have been lost to follow‐up. In all patients, arginine levels were above the target of 200umol/L virtually throughout follow‐up. Arginine and ammonia levels were both seen to increase steeply from baseline during metabolic decompensations. B, Regular sodium benzoate commenced; P regular sodium phenylbutyrate commenced; * metabolic decompensation

Figure 1 illustrates ammonia and arginine levels against age. Patients with the highest detectable ammonia levels in the context of metabolic decompensations were the ones who did not survive to adulthood (Patients 1.2 and 1.3). All patients had arginine levels that were consistently above 200 μmol/L. All patients had some difficultly adhering to protein restriction, and even during periods of good adherence, arginine levels remained elevated. Metabolic decompensations were associated with increase in arginine and ammonia levels as compared to baseline.

Elevated guanidino compounds were detectable in plasma and urine in all three patients for whom this was tested, and elevated alanine aminotransferase was seen in all patients. All patients had variably low levels of EAAs over follow‐up (Supplementary table).

### 3.5 Dietary/drug management

All patients were managed with protein‐restricted diet and were supplemented with arginine‐free amino acids with the aim of reducing plasma arginine levels <200 μmol/L while still providing adequate protein for normal growth. Total protein intake was provided as natural protein and EAA in a 40%–50%:50%–60% ratio. Sodium benzoate was used in patients with elevated baseline ammonia. Seizures were managed with antiepileptics including sodium valproate, levetiracetam and carbamazepine. In two patients, the presence of a second diagnosis complicated management. Patient 1.1 was diagnosed with type 1 diabetes mellitus. To our knowledge, this is the first published case of both conditions being present in the same individual, and we presume that type 1 diabetes was unrelated to arginase deficiency. Although there was no further dietary restriction, having to calculate carbohydrate intake for each meal as well as natural protein intake introduced significant complexity for the individual who also had learning difficulty, thereby introducing room for error and poor adherence to both insulin administration and natural protein restriction. Thus, while her insulin requirements were not high, she had poor diabetic control (HbA1c > 73 mmol/mol) but also suffered episodes of hypoglycaemia and hypo‐unawareness. We note, however, that in the follow‐up period, she had not suffered from any metabolic decompensations or ketoacidotic episodes.

Patient 2.1 had an additional diagnosis of food allergies manifesting as cow's milk protein intolerance in infancy and eosinophilic oesophagitis in later life. It was the symptoms of cow's milk protein intolerance that led to her being investigated and subsequently diagnosed with arginase deficiency. In practical terms, cow's milk protein avoidance did not confer any additional dietetic restriction since she tolerated switching to an elemental formula well. However, in adolescence, she developed eosinophilic oesophagitis causing recurrent vomiting and nearly monthly metabolic decompensations needing hospitalisation for intravenous fluids and ammonia scavengers. She responded to a short course of oral budesonide treatment; however, in the long term, exclusion of allergenic food groups such as dairy, soya, egg and wheat was felt to be too restrictive for her diet and likely to compromise her growth.

### 3.6 Utility of GAITRite assessments in patient monitoring

**FIGURE 2:**
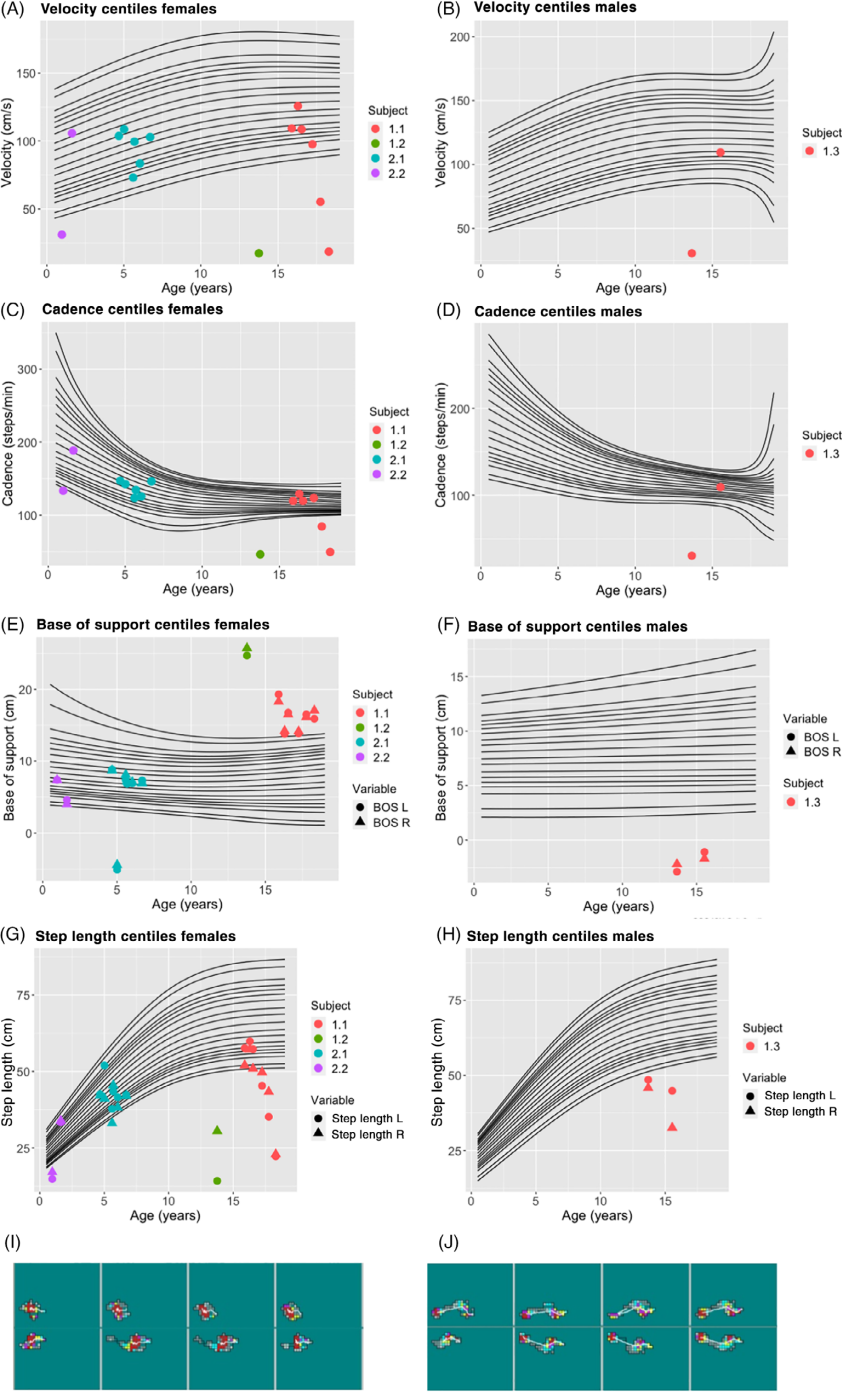
Gait velocity (A,B), cadence (C,D), base of support (E,F) and step length (G,H) measured using GAITRite system plotted on paediatric centiles. Patients 1.1, 1.2, 2.1 and 2.2 plotted on female centile curves and Patient 1.3 on male centile curves. Centile extremes correspond to the 0.5th and 99.5th centiles. Footstep analysis for Patient 1.1 before (I) and after (J) Achilles tendon release surgery demonstrating significant improvement of gait symmetry and contact surface area

Our patients had differing clinical presentations. In the family with two siblings who were asymptomatic at diagnosis (Patients 2.1 and 2.2), neurological examination was normal initially. Serial evaluation of these siblings using GAITRite revealed gait abnormalities over time. Patient 2.1 was early detected with gait abnormality at the age of 5 years in the form of negative base of support and asymmetrical step length, after being on treatment for 2.5 years (Figure 2E,G). A therapeutic programme of stretches and strengthening was introduced, and progress was monitored. Although Achilles tendon tightness and the range of movement improved quickly, the child remained unstable (asymmetrical step length). For Patient 2.2, commencement of sodium benzoate and protein restriction resulted in improved arginine levels (Figure 1) and GAITRite variables in the first 2 years of life (Figure 2A,C,G). Following emigration overseas and lost to follow up for 5 years, both siblings returned with significant neurodisability and were wheelchair‐bound. Formal assessment of gait was impossible as they were unable to walk.

The siblings of Family 1 (Patients 1.1, 1.2 and 1.3) were already significantly symptomatic at diagnosis, and their gaits were invariably abnormal. GAITRite assessments demonstrated marked differences in base of support among the siblings. These ranged from a wide‐based gait in the female siblings (Patients 1.1 and 1.2) to a negative base due to scissoring of the lower limbs in the male sibling (Patient 1.3; Figure 2E,F). In the case of Patient 1.2 the finding of a wide base of support suggests ataxia and correlates well with neuroradiological appearances indicating cerebellar atrophy. Conversely, a negative base of support suggests a scissoring gait, that is, a predominance of spasticity which is in keeping with extensive cortical atrophy seen in the brain MRI of Patient 1.3, although cerebellar atrophy was also present in this patient.

It is likely that this is due to varying degrees of corticospinal and cerebellar involvement. A combination of low gait velocity, reduced cadence and step length was also seen. In this family, the degree of neurological abnormality at baseline did not correlate with plasma arginine levels. The oldest sibling, Patient 1.1 (who had the highest average arginine levels), had the least marked changes in gait parameters as compared to her siblings. She was initially able to walk independently and had marked dynamic tone that increased when against gravity. The footstep patterns showed she was unable to put her right heel to the floor and helped guide the decision to perform Achilles tendon release. Velocity was decreasing and her step length was very asymmetrical (Figure 2G). Post‐operatively, her step lengths, although very short, became symmetrical and her gait improved (Figure 2I,J). Her younger siblings (Patients 1.2 and 1.3) both showed cogwheel/rigid tonal changes at baseline. There was evidence of improvement in Patient 1.3 after starting therapy despite suffering the setback of an acute decompensation. Some months later, when he had regained mobility, the GAITRite recorded an improvement in velocity and cadence although step length and base of support remained clearly outside the expected range (Figure 2B,D,F,H).

## 4 DISCUSSION

Arginase deficiency is normally diagnosed in early childhood with symptom onset between the ages of 2 and 4 years.^15^ In our cohort, the range of age of onset was 0.16–9 years, and the latency of diagnosis from the time of symptom onset ranged from birth in the case of the sibling of a child known to be affected to 11.5 years, supporting that diagnosis can be missed for many years. The diagnosis may not be suspected due to the condition being mislabelled as cerebral palsy.^5^, ^14^ It is important to clarify the perinatal history and recognise that neurological impairment is progressive. Classically, hyperammonaemic episodes are not as prominent in arginase deficiency compared to other urea cycle disorders, but in our cohort, they were seen frequently. Protein modelling has revealed that pathogenic missense mutations often affect residues in key positions that interact with an Mn^2+^ group within the enzyme's active site. The only genotype–phenotype correlation is that truncating mutations (as seen in our cohort) are associated with greater disease severity.^8^

Dietetic management is based upon a low natural protein intake accompanied by EAA supplementation. This diet is extremely restrictive. When applied to our patient cohort, adherence was poor in two patients, and despite good adherence in the other four, mean arginine levels were consistently elevated and EAA levels were low. Poor adherence to diet or insufficient EAA supplementation may lead to a chronically catabolic state and consequently poor growth.

Previous reports have suggested that stabilisation and even improvement in neurological function may be observed in severely affected children if arginine levels are reduced to near‐normal levels and hyperammonaemic episodes are avoided.^15^, ^16^ In this respect, the disease time course of two siblings in Family 2 (Patients 2.1 and 2.2) is instructive. These two siblings emigrated overseas over a 5‐year period, stopped taking their EAA supplements and ammonia scavengers due to lack of drug availability and had poor dietary adherence. Their disease progressed significantly over that period: from being able to walk independently to becoming wheelchair‐bound. However, when they restarted the diet and medication regime, their mobility improved. It is clear from their growth data, which demonstrated significant weight loss, that a lack of adequate overall nutrition resulted in entry into a chronically catabolic state during the 5 years they were away. It is important that metabolic multidisciplinary team impresses upon patients and their families the importance of complying with the protein restriction, EAA supplementation and ammonia scavengers.

In our cohort, GAITRite assessments were very useful in functional monitoring/detecting disease progression in a manner that is objective, quantitative and reproducible. Functional gait requires the complex interaction and coordination of most of the major muscles and joints of the lower limbs and the brain regions that control them. Decreased movement range can affect the swing through part of the cycle limiting step length, tight musculature can affect heel strike and muscular weakness and cerebellar involvement can lead to instability and affect foot placement and weight transference, thus altering the base of support. It is unsurprising that the spasticity associated with arginase deficiency produces marked abnormalities of gait parameters in affected patients. In all members of Family 1, no major improvements in gait were seen over the course of the evaluations. This is consistent with the observation that plasma arginine levels were never maintained in the desired range and EAA levels were chronically low. In our patients, GAITRite was able to detect even small changes, making it a highly sensitive modality for monitoring disease. GAITRite may represent a good functional outcome measure to detect and objectively quantify therapeutic improvement. This is particularly significant since clinical trials are currently underway to assess safety and efficacy of enzyme replacement therapy in arginase deficiency. Enzyme replacement therapy now represents an effective treatment for arginase deficiency. Since biochemical diagnosis is readily available from newborn bloodspot, arginase deficiency should be considered as a potential newborn screening target as early diagnosis might positively impact the long‐term outcomes of this patient group.

## 5 CONCLUSIONS

In our patient cohort, despite adherence to dietary interventions, mean arginine levels were still above the desired target range, hyperammonaemic decompensations still occurred and functional outcomes were generally poor resulting in significant neurodisability. New effective disease‐modifying therapies are needed. Disease progression and response to therapies could be monitored with the GAITRite system which is a sensitive marker of functional neurological status.

## CONFLICT OF INTERESTS

The authors declare that they have no conflict of interest.

## AUTHOR CONTRIBUTIONS

*Conceptualisation*: Michelle Wood, Maureen A. Cleary, and Spyros Batzios. *Data collection*/*analysis*: Nandaki Keshavan, Michelle Wood, Lucy M Alderson, and Mario Cortina‐Borja. *Manuscript writing*: Nandaki Keshavan and Michelle Wood. *Manuscript editing*: Nandaki Keshavan, Michelle Wood, Lucy M. Alderson, Mario Cortina‐Borja, Rachel Skeath, Mel McSweeney, Marjorie Dixon, Maureen A Cleary, Emma Footitt, and Spyros Batzios.

## ETHICS STATEMENT

Not required.

## INFORMED CONSENT

Informed consent was obtained from all patients for being included in the study. This article does not contain any studies with human or animal subjects performed by any of the authors.

## Supporting information


**Appendix** **S1** Supporting Information

## Data Availability

Data archiving is not mandated but data will be made available on reasonable request.
