# Supplementary material for: Clinical status, biochemical profile and management of a single cohort of patients with arginase deficiency
Source: JIMD Rep. 2021 Dec 30;63(2):123–30. doi: 10.1002/jmd2.12266 (PMC8898719; doi:10.1002/jmd2.12266)
Supplement: Supplementary file 1 — Appendix S1 Supporting Information [file JMD2-63-123-s001.docx]

**SUPPLEMENTARY DATA**

**Case Series**

**Patient 1.1**

Patient 1.1 was born at term via normal vaginal delivery to Somali parents who are second cousins. There were no perinatal complications. She developed type one diabetes mellitus requiring insulin at 18 months. There was a history of motor delay with delayed walking and noticeable toe walking at five years which became gradually worse through childhood. She had moderate learning difficulties, including problems in verbal skills, perceptual reasoning, working memory, processing speed and a full-scale IQ of 53 (<1^st^ centile) requiring considerable help in a mainstream school. Neurological examination at the age of 15 years revealed spastic diplegia: lower limb hypertonia, brisk reflexes, bilateral clonus and grade 3-4/5 power. Her posture included flexion at the hips and knees and tiptoeing due to tight gastrocnemius tendons bilaterally. She was unable to stand from a sitting position without using her arms. Despite an unstable slightly scissoring gait she was independently mobile but complained of getting tired quickly. She received a biochemical diagnosis of arginase deficiency at 15.5 years following which she was seen at our paediatric metabolic clinic and a decision was made to commence protein restriction of 1.1g/kg/day (0.8g/kg/d of natural protein and 0.3g/kg/d of EAA supplements) with the additional aim of achieving good control of her diabetes mellitus. Unfortunately, throughout, her adherence to dietary intervention and insulin was problematic. At the age of 17.3 years she was started on sodium benzoate 235mg/kg/day in view of baseline hyperammonaemia. She required ankle-foot orthoses and Achilles tendon release surgery bilaterally in order to improve her gait and at 22 years commenced on tizanidine and baclofen to manage spasticity. Her mobility deteriorated slowly requiring her to use elbow crutches to walk and a wheelchair for long distances. She however made cognitive progress and was able to go to college. At 23.5 years it was noted that she had overall poor diabetic control - HBA1c 73 mmol/mol (8.8%) and problems of hypoglycaemia and hypo-unawareness. She also developed myoclonic seizures with EEG demonstrating a background of generalised epilepsy requiring commencement of levetiracetam. To date, aged 27 years she is wheelchair dependent and has not had any hyperammonaemic decompensations.

**Patient 1.2**

Patient 1.2 was the younger sister of patient 1.1. She was born at term via vaginal delivery. She was well until 2.5 years when she developed a high stepping gait. She displayed developmental regression of motor abilities and speech. By three years she was unable to stand and had only single words in her vocabulary. She developed absence episodes from 9 years and generalised tonic-clonic seizures from 11 years and was commenced on sodium valproate. MRI head showed evidence of cerebral atrophy. She received a biochemical diagnosis based on testing following diagnosis of her sister at 11.5 years. She commenced protein restriction soon after. At 12 years she presented to hospital in status epilepticus requiring intubation and intensive care. She also became hyponatraemic and hyperammonaemic (197). Neurological examination demonstrated hypertonia in all four limbs (legs worse than arms), hyperreflexia, wasting of the muscles of the legs and significant functional disability requiring assistance will all activities of daily living and a wheelchair to walk. Due to low weight she was commenced on Paediasure feeds (equivalent to 0.5g/kg/d of protein) via nasogastric tube, and drugs included sodium benzoate 182mg/kg/day, EAA supplementation (equivalent to 0.4g/kg/d protein equivalent), and carbamazepine. She was weaned off sodium valproate. At 13.2 years she received bilateral Achilles tendon release surgery**,** hamstring and adductor releases and also fitted with ankle foot orthoses. This improved her motor ability and enabled her to pull herself out the wheelchair into a standing position and walk short distances with the aid of one person. She attended a special-needs school and had significant speech delay. At 14.8 years she presented to hospital with hyperammonaemic decompensation and seizures (highest ammonia 459, arginine 525, Ornithine 131) precipitated by constipation and vomiting. She was admitted to intensive care where she was administered maximum doses of intravenous ammonia scavengers (sodium phenylbutyrate and sodium benzoate at 250mg/kg/ day). On discharge, she was commenced on 15g EAA(0.41g/kg/d protein equivalent) in addition to Paediasure and natural protein at an additional 0.41g/kg/d, and ornithine supplements of 100mg/kg per day. At 15.4 years she re-presented in status epilepticus, hyperammonaemic decompensation (highest ammonia 345, arginine 818, ornithine 33), rhabdomyolysis causing acute kidney impairment and hepatic failure. She was intubated and admitted to intensive care where she subsequently developed profound encephalopathy. Neuroimaging showed extensive ischaemic changes to most of the brain. She later developed bowel perforation, candida peritonitis and sepsis and subsequently died due to multiorgan failure.

**Patient 1.3**

Patient 1.3 was the younger brother of patients 1.1 and 1.2. He was born by normal vaginal delivery and there were no postnatal problems. Concerns started in infancy when he displayed motor delay and commenced walking late at 17 months. At 2 years he started to toe walk. He developed generalised tonic-clonic seizures at 6 years for which he was commenced on sodium valproate with good effect. At 8 years he had hamstring and Achilles tendon lengthening surgery. Neurological examination at 13 years revealed spastic quadriplegia including hypertonia, clonus, weakness with his legs being more profoundly affected. His standing posture included flexion at the hips and knees and a tendency to weight bear on lateral borders of feet and scissoring. He needed a walking frame to mobilise. Following diagnosis of his elder sister, patient 1.1, he was found to have an elevated plasma arginine at 13.25 years and was commenced on a dietary regime comprising 1g/kg/d of natural protein and 13g EAA supplements (0.5/g/kg/d protein equivalent). At 14.8 years he presented to hospital with increased seizures, decreased consciousness and hyperammonaemia (highest ammonia 265, arginine 628, ornithine 38). CT head showed cerebral oedema with marked cerebellar hypoplasia and he was transferred to intensive care for further management. He was able to recover from this decompensation and was started on sodium phenylbutyrate 231mg/kg/day, and carbamazepine and sodium valproate stopped. He had difficulty complying to protein restriction and he continued to suffer from brief clusters of seizures. At 16.8 years he developed a second major hyperammonaemic decompensation (highest ammonia 283, arginine 602, ornithine 39 presenting to hospital with vomiting, lethargy, increased seizures and reduced consciousness. He developed status epilepticus and was intubated and transferred to the intensive care unit. CT head showed diffuse cerebral hemisphere damage and acute cerebral oedema. He developed fever and investigations revealed acute EBV viraemia. He was able to recover from the decompensation but suffered overall neurological deterioration including spasms, agitation, self-harm (scratching self), temperature dysregulation and possible visual loss as he did not appear to fix and follow anymore although his visually evoked potentials were normal. He was maintain on a dietary regime of total 1.1g/kg/d of protein of which half was EAA supplements and half was from gastrostomy feeds. He was commenced on baclofen, and ornithine supplementation in addition to regular oral sodium benzoate and sodium phenylbutyrate. At 18.5 years he was wheelchair bound, doubly incontinent, lacked speech, and was entirely gastrostomy fed.

**Patient 2.1**

Patient 2.1 was born at term via emergency Caesarean section for failure to progress after normal pregnancy to Somali parents who were not known to be consanguinous. No special care was required postnatally. She was noted to have vomiting and reflux during the first three months of life and appeared to have a poor diet and loose stools during the first year of life. Routine blood tests were performed at 1.5 years when she was admitted for an elective periumbilical hernia repair which showed deranged liver function (ALT 105, Alb 21, INR 2.4) and iron deficiency anaemia (Hb 49, ferritin 2). A diagnosis of cow’s milk protein intolerance was made and iron supplementation commenced along with a switch to a fully hydrolysed formula. Plasma amino acid analysis demonstrated hyperargininaemia and erythrocyte arginase enzyme activity was absent. Targeted genetic testing later confirmed homozygosity for c.646_649delCTCA p. Leu216Afs*4 in *ARG1*which has been previously reported (Wu et al. 2013). At 2 years she was commenced on a low-protein diet of 1g/kg/day. Her neurological examination was normal although her speech was slightly delayed: she was only able to speak a few words in Somali.

At 2.5 years she was commenced on sodium benzoate and sodium phenylbutyrate and EAA supplements which were optimised over several months.

At 4.5 years her parents noted that she started to toe-walk and a few months later she started complaining of leg pain when walking. Examination showed evidence of early spasticity developing in the tendons of the wrist and her Achilles tendons. She was also noted to have mild left leg weakness and clonus bilaterally (left more than right). Due to progression of spasticity as evidenced by brisk leg reflexes, clonus and muscle wasting, she received regular physiotherapy with good effect. Her arginine levels were above the target of 200mmol/L and she had difficulty gaining weight as her appetite was poor but she remained compliant to protein restriction and continued to take her EAA supplements. Her total protein intake was 1g/kg/d of which 0.45g/kg/d was natural protein and 0.55g/kg/d was EAA supplement. She was lost to follow-up between the ages of 7 and 12 years since the family moved overseas where all her medications were discontinued due to unavailability. On return at 12 years the family recounted that she physically deteriorated around the age of 8 years to the point of being unable to walk. On reassessment she looked thin, had evidence of spastic quadriplegia with a scissoring posture of legs, increased tone, brisk reflexes and muscle wasting in all four limbs. There was marked tightness of hip extensors/abductors, Achilles tendons, limited rotation of hips, tight hamstrings resulting in mobility limited to crawling at home or needing to be carried by her parents. She was fitted with ankle-foot orthoses to improve her tip-toeing. Her parents also expressed concerns about deterioration in her cognitive ability and needed admission to a special school. At 12.3 years she suffered a first hyperammonaemic decompensation (maximum ammonia 173 umol/L) requiring 8 days of hospitalisation following an intercurrent febrile illness. At 13.1 years she received tendon lengthening surgery to both feet but this made little difference to her functional ability. Between the ages of 13 – 15 years she suffered 5 hyperammonaemic decompensations (maximum ammonia levels: 117, 254, 161,131,143 umol/L) precipitated by vomiting requiring hospitalisation for intravenous ammonia scavengers and commencement of regular sodium benzoate at 260mg/kg/day. Paired plasma arginine and ornithine values were not available for these episodes which were managed in the local hospital. And impedance study showed a degree of mild reflux that was within normal limits and upper GI endoscopy showed evidence of eosinophilic oesophagitis which responded histologically to oral budesonide and after 3 months her symptoms improved considerably and she was able to wean off budesonide. To date, aged 16 years she continues to require a wheelchair to mobilise and a standing frame but was able to take her Year 10 exams at school.

**Patient 2.2**

Patients 2.2 is the younger sister of patient 2.1. She was born by normal vaginal delivery after a normal pregnancy with a birthweight of 3.9 kg. Postnatally her arginine levels were measured and found to be elevated. Genetic testing showed homozygosity for the same pathogenic variant as for her older sister. She was breastfed initially and following confirmation of the biochemical diagnosis, she was given reduced protein feeds and EAA supplements. Her total protein intake was managed at ~1.4g/kg/d of which 0.67g/kg/d was natural protein and 0.73g/kg/d was EAA supplements. She was noted to vomit frequently after feeds. At 3 months she was commenced on sodium benzoate. Arginine levels improved on this regime. Her development was normal. Between the ages of two and seven years the family emigrated overseas and her medications were stopped due to unavailability. On return at 7 years her parents recounted that she had stopped walking at age 3 years. Like her sister she also developed spasticity and appeared thin. Neurological examination revealed increased leg tone with brisk reflexes, very tight Achilles tendons, ankles in equinovarus posture, flexion of hips and knees when standing, reduced muscle power of the legs all contributing to reduced independent mobility requiring use of a wheelchair. She also had learning difficulties, delayed receptive and expressive speech and poor concentration. She was recommenced on protein restriction at ~9g/kg/d of which 0.6g/kg/d was from low protein food/feeds and 0.31g/kg/d was EAA supplementation.

At 7.3 years she suffered her first hyperammonaemic decompensation (highest ammonia 80umol/L) requiring treatment with sodium benzoate. At 8.5 years she underwent bilateral Achilles tendon lengthening, and releases of the plantar fascia bilaterally, left abductor hallucis, left calcaneofibular tendon and was fitted with ankle-foot orthoses.

Her surgery had a significant positive impact on her progress and enabled her to walk initially with a frame but then independently albeit with an asymmetrical gait attributed to a 2 cm leg length discrepancy. She displayed improved educational development and academic performance in mainstream school but needed 1:1 support. She suffered 3 hyperammonaemic decompensations (highest ammonia levels 99, 200, 195 umol/L) between the ages of 10-11 years precipitated by vomiting, but was managed with intravenous fluids and ammonia scavengers in the local hospital. Paired arginine and ornithine data are unavailable for these episodes.

**Patient 3**

Patient 3 was born at 38 weeks gestation via normal vaginal delivery to Pakistani parents who are second cousins. He was noted to be difficult to feed postnatally with frequent vomiting resulting in weight loss in early infancy. He was diagnosed with cow’s milk protein intolerance. His early milestones were within normal limits but he was noted to have an unusual gait pattern with foot in-turning and toe walking at 6 years resulting in difficulty in using the stairs and running. He also developed learning difficulties, struggled with fine motor skills and manipulation of objects, and neuropsychological assessment revealed a full IQ score of 44 (IQ <0.1 centile) which included indicators of difficulty with working memory, processing speed and overall intellectual ability and was placed in a special school. He had his first generalised seizures, including absence episodes at the age of 10 years. EEG showed multifocal epilepsy. He was commenced on sodium valproate which controlled seizures well and sodium benzoate 150mg/kg/day in view of persistently elevated baseline ammonia levels. Plasma amino acids showed hyperargininaemia and genetic testing later showed homozygosity for c.93_93delG;p.Arg32Glufs*16 which has been previously reported (Hertecant et al. 2009). He was also managed on a dietary regime of a total protein restriction of 1g/kg/day of which 0.6g/kg/d was natural protein and 0.4g/kg/d was EAA supplementation. He had 3 hyperammonaemic decompensations (highest ammonia levels 180, 90, 163 umol/L) requiring intravenous ammonia scavengers between the ages of 12 - 13 years secondary to viral infections managed in the local hospital. Presently he continues to mobilise independently but with hypotonia, tight Achilles tendons and clonus in both legs indicating spastic diplegia.

**Supplementary table 1: Biochemical data.**

| ID | Mean NH3 | Mean Arginine | Mean Ornithine | Mean Threonine | Mean Leucine | Mean Isoleucine | Mean Valine | Mean Lysine | Mean Phenylalanine | Mean Tryptophan | UGAA/ Cr ratio | Plasma GA | Highest ALT |
| --- | --- | --- | --- | --- | --- | --- | --- | --- | --- | --- | --- | --- | --- |
| 1.1 | 40.4 | 572.4 | 29.48 | 54.46 | 54.24 | 25.90 | 106.48 | 81.11 | 36.47 | 28.20 | ND | ND | 134 |
| 1.2 | 216.8 | 438.3 | 29.24 | 122.80 | 67.23 | 34.51 | 143.20 | 116.59 | 29.91 | 22.76 | ND | ND | 454 |
| 1.3 | 165.1 | 355.5 | 39.00 | 61.04 | 27.91 | 124.59 | 124.59 | 108.40 | 27.04 | 21.80 | ND | ND | 309 |
| 2.1 | 38.8 | 368.4 | 34.98 | 62.06 | 57.56 | 29.85 | 120.34 | 96.26 | 41.38 | 29.28 | 184 | 2.9 | 832 |
| 2.2 | 39.5 | 334.1 | 28.00 | 55.26 | 60.98 | 31.95 | 129.59 | 93.45 | 39.83 | 28.58 | 272 | 2.3 | 443 |
| 3 | 84.9 | 471 | 34.35 | 47.58 | 54.99 | 30.96 | 105.00 | 71.87 | 32.14 | 15.46 | 222 | 6.2 | 125 |

Reference ranges: Ammonia (NH3) (<40 umol/L), Arginine (40-120 umol/L), Ornithine (25-120umol/L), Threonine(70-220umol/L), Leucine (65-220umol/L), Isoleucine (26-100umol/L), Valine (90-300umol/L), Lysine (100-300umol/L), Phenylalanine (35100umol/L), Tryptophan(30-80umol/L), Urine Guanidinoacetate/ creatinine(UGAA/Cr) ratio (10-100 umol/mmol), Plasma Guanidinoacetate (GA) (0.8-3.1 umol/L), alanine aminotransferase (ALT) (10-45 U/L). Red indicates above upper limit of reference range, blue indicates below lower limit of reference range. ND, not done.

**Supplementary Figure 1**

**
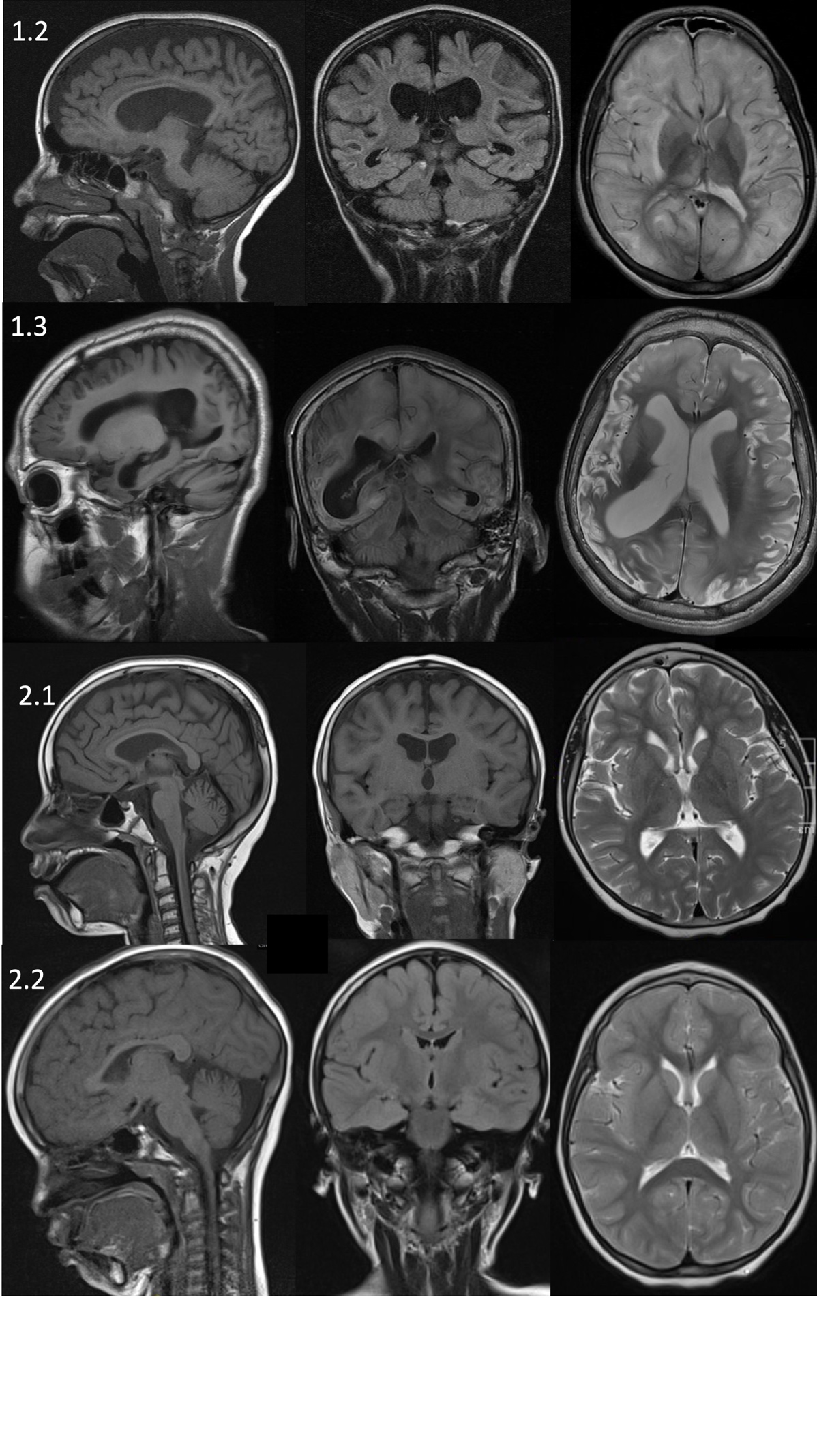
**

**Supplementary Figure 1** Neuroimaging for patients 1.2,1.3, 2.1, 2.2. Sagittal T1, coronal T1 and transverse T2 images are included for each patient. All cases demonstrated generalised cerebral and cerebellar atrophy. Patient 1.3 also had pontocerebellar hypoplasia and bilateral asymmetric regions of cortical atrophy.

**Supplementary Figure 2**


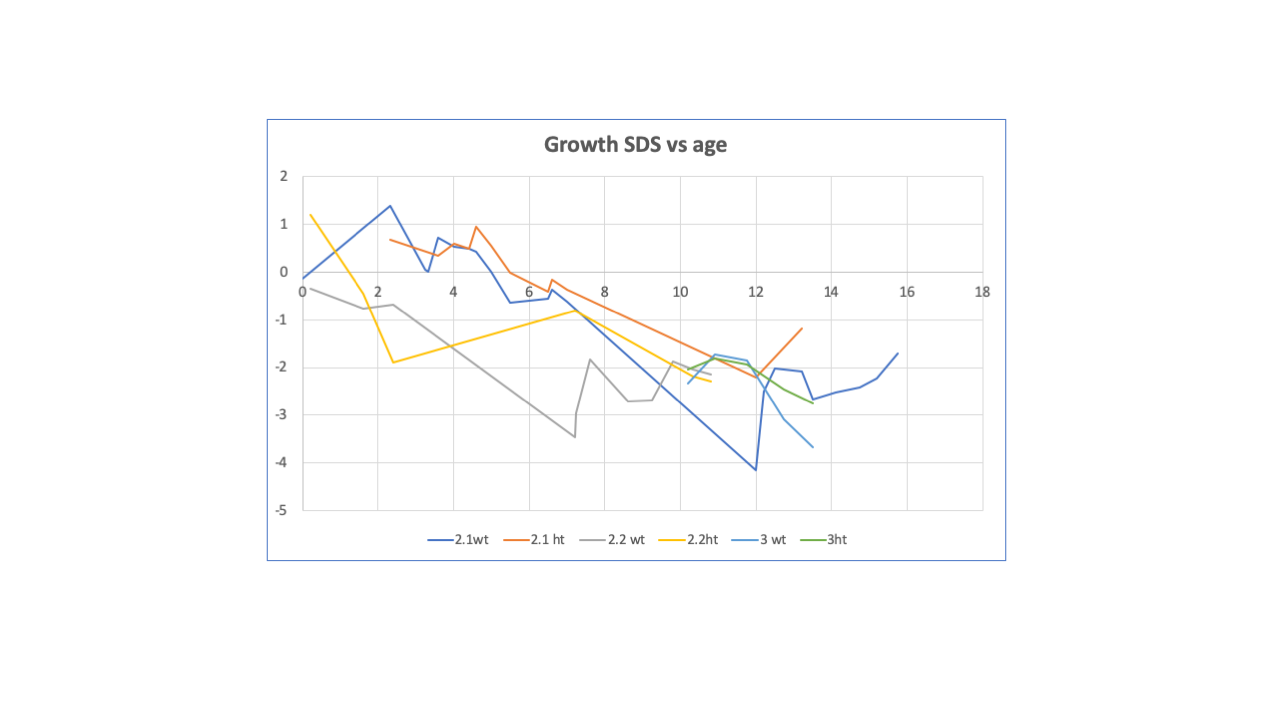


**Supplementary Figure 2** Growth data is shown for patients 2.1, 2.2 and 3 to demonstrate height (ht) and weight (wt) standard deviation scores (SDS) vs age (years). For patients 2.1 and 2.2 there are no data points between the ages of 7-12y and 2-7y respectively as they have been lost to follow up.
